# Should Priority Setting Also Be Concerned About Profound Socio-Economic Transformations? A Response to Recent Commentary

**DOI:** 10.15171/ijhpm.2017.85

**Published:** 2017-07-17

**Authors:** Brayan V. Seixas, Craig Mitton, Marion Danis, Iestyn Williams, Marthe Gold, Rob Baltussen

**Affiliations:** ^1^ School of Population and Public Health, University of British Columbia, Vancouver, BC, Canada.; ^2^ National Institutes of Health, Bethesda, MD, USA.; ^3^ University of Birmingham, Birmingham, UK.; ^4^ New York Academy of Medicine, New York City, NY, USA.; ^5^ City College, New York City, NY, USA.; ^6^ Radboud University Medical Center, Nijmegen, The Netherlands.

In his comment^1^ on the editorial “Global Developments in Priority-Setting in Health” authored by Baltussen et al,^2^ Dr. Ted Schrecker provides a useful summary of the main reasons underlying the current degree of scarcity in healthcare systems worldwide. Indeed, measures of fiscal austerity, rising rates of social inequality and practices of fiscal evasion, among other things, have certainly contributed to the allocation of financial resources to public services at levels far below what we could have in a more just and cooperative world. Yet, what Dr. Schrecker misses in his paper is that working on the development of better strategies of priority-setting to allocate resources does not necessarily mean endorsing the *status quo.* This is a false polarization that, besides highlighting non-existent differences, obscures important similarities between progressive public health researchers.

First, there seems to be no need to comment on all the evidence on how the health of populations is shaped by macroeconomic policies. The growing body of consistent evidence on this topic^3,4^ has made it already a paradigmatic (in a Kuhnian sense^5^) recognition in the field of public health, albeit with no absence of criticisms.^6^ Schrecker’s diagnosis is accurate in its denunciation of contemporary market-based economies, in which governments, and consequently the poorer, are allocated ever-decreasing portions of the global wealth. Here, we are in complete agreement: our societies are operating with socio-economic structures that appropriate the wealth produced by the majority of the people in favour of a small minority. We by no means take this for granted.

However, let us focus on some of the imprecisions espoused by Dr. Schrecker. He points out the “narrowness and moral precariousness”^1^ (p. 1) of the predominant discourse on priority-setting. His argument seems to rely on the assumption that by advancing strategies for priority-setting, we are endorsing this current state of affairs. In fact, he explicitly states that by acknowledging the impossibility of resourcing all possible demands on healthcare, we are assuming “that the current budget is the appropriate one”(p. 2).^1^ In fact, in the vast literature on priority-setting, it is difficult to find passages where any authors defend the contemporaneous rate of investment on healthcare as being optimal. After all, is it even possible to determine an ideal level of spending in healthcare? The question remains unresolved. What is indeed normally assumed is that the current budget is the one that decision-makers will have to work with for that fiscal period, regardless of one’s view of the political process of setting the overall funding allocation. The research community dedicated to advancements in the field of priority-setting is, in this sense, mostly concerned with helping policy-makers to make better choices, ensuring the available resources are allocated in a fashion that not only takes into account the economic concepts of efficiency and equity in health, but also that considers the importance of public engagement and transparency.

Further, Dr. Schrecker argues that these efforts of advancing the practices of priority-setting and decision-making tend to over-shadow the upstream political causes underpinning the level of scarcity observed in healthcare systems. However, there are two major flaws in this rationale.

First, these apparently opposing positions are not necessarily mutually exclusive. Public health researchers can at the same time pursue methodologies for better priority-setting and decision-making and condemn the ills wrought by contemporary market-based economies on population health. Dr. Schrecker even provocatively states that researchers are taking part in decisions with “disabling if not homicidal impact”^1^ (p. 2). But what is our role in this struggle? Should we only keep protesting and vocalizing our anger and frustration until our efforts to ‘interrogate scarcity’ bring about some fundamental change in the western socio-economic structure and its dynamics? Or maybe until the pharmaceutical companies decide to charge fair prices, or governments decide to restrain them in concert with more welfare-oriented governance? What happens with all the decisions that need to be made in the meantime? Without adequate processes of decision-making, certainly many more people will suffer “disabling if not homicidal impact” through our *not* maximizing use of the available resources. As researchers, focusing solely on long-term transformation to the socio-economic order may leave us morally intact but it also renders us redundant in the more immediate decision-making arena.

The second major weakness of the argument relies on a missing middle premise: that it is possible to live in a world without scarcity of resources. Besides being an idea with no supporting evidence, this is also naïve in its disregard for the ‘inevitability of hard choices’ in a world with necessarily limited resources and potentially infinite demands.

In summary, instead of emphasizing minor differences among serious members of the research community, we could combine efforts towards important social transformations. Whereas some of us can work more intensely in the front-line of long-term changes to upstream social dynamics, denouncing the primary causes of the current degree of scarcity imposed upon governments and citizens, others can focus on guaranteeing that we provide the best possible health and healthcare to our populations right now by encouraging the best use of the scarce resources available at the present. Above all, academics who dream of a more solidary and just world need to make sure we are all taking efforts to improve the health of populations, both in the short and long-term, both ideally and pragmatically.

## Ethical issues

Not applicable.

## Competing interests

Authors declare that they have no competing interests.

## Disclaimer

The views expressed here are those of the authors and not a reflection of the policies of the institutions where they work.

## Authors’ contributions

BVS wrote the first draft which was reviewed CM, MD, IW, MG, and RB who each provided substantive editing leading to the final accepted version.

## Authors’ affiliations

^1^School of Population and Public Health, University of British Columbia, Vancouver, BC, Canada. ^2^National Institutes of Health, Bethesda, MD, USA. ^3^University of Birmingham, Birmingham, UK. ^4^New York Academy of Medicine, New York City, NY, USA. ^5^City College, New York City, NY, USA. ^6^Radboud University Medical Center, Nijmegen, The Netherlands.
